# Are levator hiatal dimensions in mid-pregnancy associated with mode of delivery?

**DOI:** 10.1007/s00192-022-05111-x

**Published:** 2022-03-01

**Authors:** Frøydis Folvik Bjerkholt, Maria Øyasæter Nyhus, Seema Mathew, Ingrid Volløyhaug

**Affiliations:** 1 Faculty of Medicine and Health Sciences, Norwegian University of Science and Technology, Trondheim, Norway; 2 Department of Clinical and Molecular Medicine, Norwegian University of Science and Technology, Trondheim, Norway; 3 Department of Obstetrics and Gynecology, St. Olavs University hospital, Trondheim, Norway

**Keywords:** Birth injury, Childbirth, Delivery, Pelvic floor, Pregnancy, Ultrasound

## Abstract

**Introduction and hypothesis:**

Slow progress of labour is a risk for operative delivery. Smaller levator hiatal dimensions are possible risk factors for slow progress and operative delivery. Our aim was to explore associations between hiatal dimensions antenatally, duration of second stage of labour and mode of delivery.

**Methods:**

Prospective cohort study of 65 nullipara examined at 20 weeks gestation and 6 months postpartum. Levator hiatal anteroposterior diameter and area were measured using 2D/3D transperineal ultrasound and compared between women with normal vaginal delivery and operative delivery (vacuum or caesarean) using *t*-test and with Spearman’s rank to explore correlations with duration of second stage. ROC analysis established a cut-off for high risk of operative delivery.

**Results:**

Two-dimensional anteroposterior diameter and 3D hiatal area at rest were smaller in women with operative delivery than with normal delivery, 5.0 cm vs. 5.7 cm, *p* = 0.007 and 18.5 cm^2^ vs. 14.9 cm^2^, *p* < 0.001. From the ROC curve for 2D anteroposterior diameter, a cut-off of 5.6 cm, (sensitivity = 0.94, specificity = 0.63) and for 3D hiatal area a cut-off of 17.6 cm^2^ (sensitivity = 0.94, specificity = 0.65) predicted operative delivery. We found inverse correlations between second stage of labour and anteroposterior diameter at rest, r = −0.330, contraction, r = −0.365, area at rest, r = −0.324, and contraction, r = −0.521, all *p* < 0.05.

**Conclusions:**

Smaller hiatal dimensions at 20 weeks gestation were associated with longer second stage of labour and increased risk of operative delivery in nullipara. A 2D anteroposterior hiatal diameter < 5.6 cm and 3D hiatal area < 17.6 cm^2^ at rest imply increased risk of operative delivery.

## Introduction

Slow progress of labour increases the risk of operative delivery [1, 2]. There are several known risk factors for unplanned operative delivery including antenatal variables such as maternal body mass index (BMI), height, age and estimated fetal birthweight [3–6]. In addition, intrapartum parameters such as induction of labour, Bishop score, oxytocin augmentation, stage and position of the fetal head are associated with risk of operative delivery [7–9].

During the process of labour, the fetal head exerts a large force on the pelvic floor as the fetus progresses through the birth canal. The levator ani muscle (LAM) has to stretch around three times its original length, making it a non-negligible barrier for a normal vaginal delivery [10]. A few studies have attempted to describe relationships between levator hiatal dimensions antepartum and mode of delivery [11–13]. Results have been somewhat inconclusive, but indicate that women with smaller hiatal dimensions on 3D/4D ultrasound have a tendency toward longer second stage of labour, which in turn could cause higher rates of operative delivery [11–13]. The role of levator morphology in birth mechanics is not fully understood, and whether smaller hiatal dimensions is an independent risk factor for operative delivery needs further investigation. If hiatal dimensions in early pregnancy could be used to determine increased risk of operative delivery, this could improve the selection of women for low- or high-risk delivery units and contribute to planning the level of monitoring during delivery. Three-/four-dimensional ultrasound requires post-processing of ultrasound volumes, and if 2D technique can be applied, this will increase the clinical utility of ultrasound.

The purpose of this study was therefore to explore associations between hiatal dimensions measured antenatally and mode of delivery, in particular the value of 2D ultrasound measurements. We also explored the association between duration of second stage of labour and hiatal dimensions.

## Methods

This was a prospective cohort study of 65 Caucasian nullipara examined at Trondheim University Hospital between 1 January 2017 and 30 June 2018. The women in this cohort were part of a larger study where the primary aim was to determine intra- and interrater reliability and agreement for ultrasound measurements of pelvic floor muscle contraction and correlation between ultrasound and palpation for assessment of pelvic floor muscle contraction [14]. The study sample size was based on power calculations for the primary outcome in the parent study, requiring 65 pregnant women. Postpartum data of this cohort have not been analyzed previously. Eligibility criteria were age >18 years, ability to consent, singleton pregnancy, no previous deliveries and being fluent in Norwegian or English. The study was approved by the Regional Committee for Medical and Health Research Ethics (REK midt 2015/1751) and registered in clinicaltrials.gov with identifier NCT03064750.

The women were enrolled at the 18-week routine ultrasound examination and examined at 20 weeks gestation and 6 months postpartum. All were low-risk nullipara planned for a normal vaginal delivery. Details regarding height, weight, age, infant birthweight, induction of labour, duration of active second stage of labour, gestational length, use of epidural analgesia and mode of delivery were collected from electronic patient journals several weeks to months after delivery. Mode of delivery was categorized into normal vaginal and operative delivery, the latter including vacuum, forceps and caesarean section after the start of labour.

The women were examined in the supine position in a gynecological examination chair, with knees and hips semi-flexed and abducted. Bladder and bowel were emptied prior to the examination, and the women were thoroughly instructed in how to perform pelvic floor contraction and maximal Valsalva manoeuvre.

A 3D/4D ultrasound examination of the pelvic floor was performed by one of three experienced clinicians (M.Ø.N., S.M., I.V.) using a Voluson GE S10 or E8 (GE healthcare, Zipf, Austria) device with a RAB 4–8-MHz curved array transducer, and the acquisition angle was set at 85°. The ultrasound examination was performed with the transducer in the midsagittal plane and the following volumes were recorded: from rest to maximum pelvic floor muscle contraction and from rest to maximal Valsalva manoeuvre [15].

**Fig. 1:**
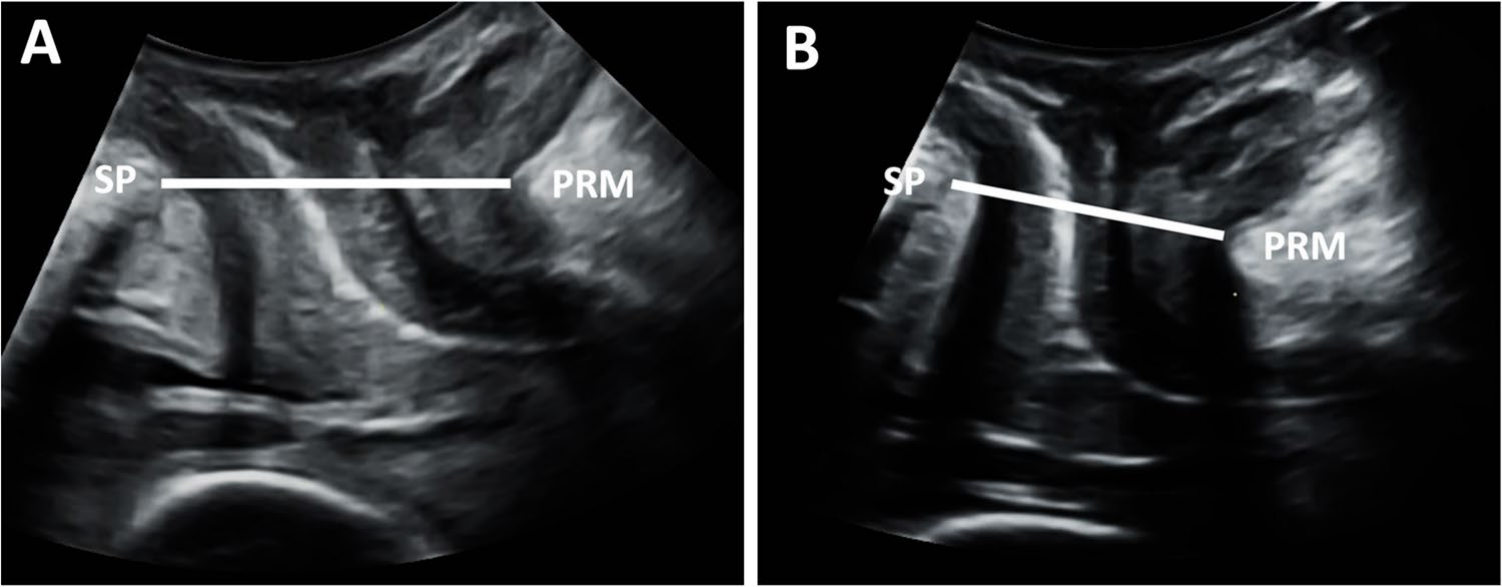
Dimensions of the levator hiatus at 20 weeks gestation. The 2D anteroposterior diameter is measured from the symphysis pubis (SP) to the medial portion of the puborectalis muscle (PRM) at rest (**A**) and contraction (**B**)

**Fig. 2:**
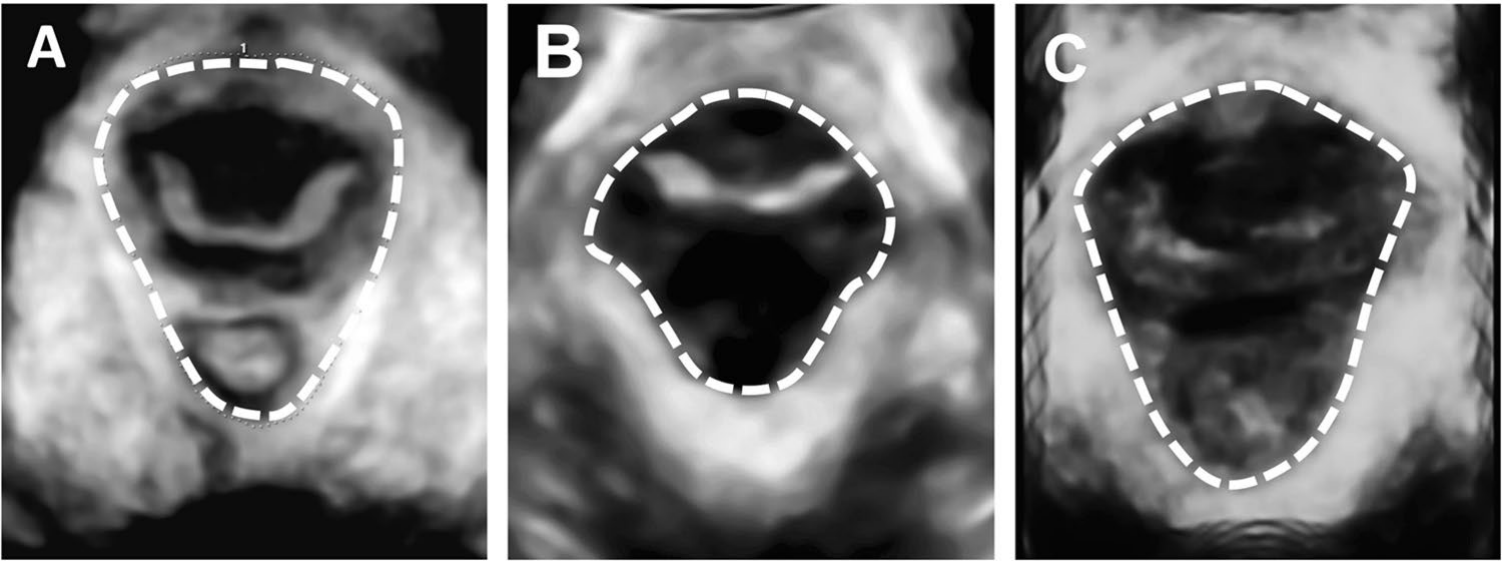
Area of the levator hiatus at 20 weeks gestation. The 3D area is measured by tracing the levator hiatus in the rendered 3D volume during rest (**A**), contraction (**B**) and at Valsalva (**C)**

Offline analysis of the ultrasound volumes was performed 6–12 months after the ultrasound scan by one examiner (M.Ø.N.) using 4Dview Version 14 Ext.0 (GE Healthcare, Austria) software, blinded to all clinical data. Volumes from the 20 weeks gestation scan were used to measure the anteroposterior diameter of the hiatus (from the distal point of the symphysis pubis to the proximal point of the puborectalis muscle) at rest and contraction in the 2D mid-sagittal image; see Fig. 1. The rendered 3D volume was used to measure the levator hiatal area at rest, during contraction and Valsalva, as previously described; see Fig. 2 [15]. These measures have previously been demonstrated to have high inter- and intrarater reliability in women in this study population [14, 15]. Volumes from the postpartum scan were used to identify LAM macrotrauma and anal sphincter injury according to standardized criteria [15, 16].

Statistical analysis was performed using SPSS version 26 (SPSS Inc., Chicago, IL). Normality of the continuous variables was assessed using histograms, boxplots and QQ plots. The independent samples *t*-test and chi-square test were used to investigate differences in hiatal dimensions and obstetrical data between women with operative (caesarean or vacuum) and normal vaginal delivery. Other possible confounders (maternal age, weight, height, infant birthweight) were not associated with mode of delivery or hiatal dimensions and therefore not included in the analysis.

We used receiver-operator curve (ROC) analysis to define a cut-off value of hiatal dimensions for women at high risk of operative delivery. Spearman’s rank correlation analysis was used to test the association between hiatal dimensions and duration of second stage of labour. The closer the correlation coefficient is to 1, the stronger the correlation: > 0.3 = weak correlation; > 0.5 = moderate correlation; > 0.7 = strong correlation [14]. Women registered with duration of active second stage of 0 min were eliminated from analyses of duration of active second stage since they were all first stage caesarean section cases.

Level of statistical significance was set at 5% for all tests.

## Results

In total, 56/65 (86%) women completed the study. One participant declined further participation, five did not attend the follow-up, and three were excluded because of missing ultrasound volumes. There were 40 (71.4%) normal vaginal deliveries and 16 (28.6%) operative deliveries. Among the operative deliveries, seven (12.5%) were caesarean sections and nine were vacuum-assisted (16.1%). There were no forceps deliveries. Eleven (19.6%) women were registered with levator ani macrotrauma 6 months postpartum, two (3.6%) had anal sphincter injury, 34 (60.7%) had epidural, and six (10.7%) had induction of labour. In the operative delivery group, epidurals were more frequent. There were no intrauterine foetal deaths or preterm deliveries and the mean (95% CI) gestational age was 281.7 (279.7–283.7) days. Table 1 shows background characteristics grouped by mode of delivery. No significant difference in BMI, height, weight, maternal age or infant birth weight was found between women with normal vaginal and operative delivery.

**Table 1:** Obstetrical data of nulliparous women at 20 weeks gestation grouped by delivery mode with comparison between the groups (independent samples *t*-test)

	NVD, *n* = 40* Mean (95% CI)	OD *n* = 16 Mean (95% CI)	*p* value
Maternal age (years)	29.6 (28.4–30.8)	31.2 (28.7–33.7)	0.193
Maternal height (cm)	167.8 (165.7–170.0)	165.4 (162.7–168.2)	0.203
Maternal weight (kg)	66.7 (62.9–68.7)	63.1 (66.2–72.2)	0.721
Maternal BMI (kg/m^2^)	23.3 (22.3–24.4)	24.5 (22.4–26.6)	0.265
Infant birthweight (g)	3469.8 (3359.8–3579.7)	3628.1 (3444.5–3811.8)	0.126

NVD = normal vaginal delivery; OD = operative delivery; AP = anteroposterior

*One case missing for data on height and weight. *n* = 39

**Fig. 3:**
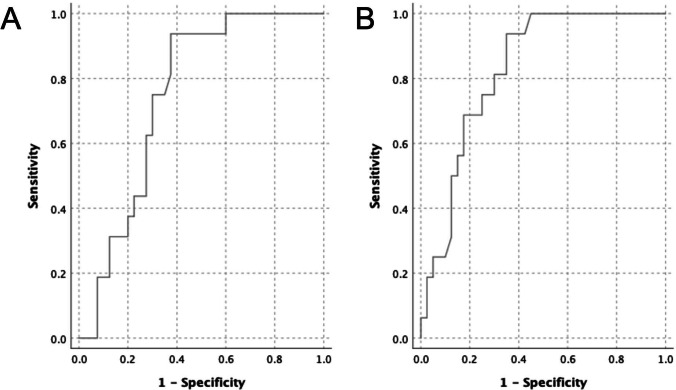
ROC curve of the predictive performance of 2D levator hiatal anteroposterior diameter at rest (**A**) and 3D levator hiatal area at rest (**B**) for operative delivery in nulliparous women at 20 weeks gestation

Table 2 shows hiatal dimensions at 20 weeks gestation grouped by mode of delivery. We found a significant difference in 2D levator hiatal anteroposterior diameter at rest between women with normal vaginal delivery and operative delivery. The mean diameters were 5.7 cm and 5.0 cm, respectively, *p* = 0.007. A significant difference was also found for 3D hiatal area at rest, 18.5 cm^2^ vs. 14.9 cm^2^ (*p* < 0.001). A ROC curve was constructed to test the predictive performance for operative delivery of 2D levator hiatal anteroposterior diameter at rest; see Fig. 3A. The area under the curve (AUC) was 0.75 (*p* = 0.004, 95% CI = 0.62–0.87). The cut-off of the 2D anteroposterior diameter was set to 5.6 cm, giving a sensitivity of 0.94 and a specificity of 0.63 to predict operative delivery. In our population, 30 (53%) women had an anteroposterior diameter < 5.6 cm. For comparison, a similar ROC curve was constructed for 3D hiatal area at rest; see Fig. 3B. The AUC was 0.83 (*p* < 0.001, 95% CI = 0.72–0.93). The cut-off area at rest was set to 17.6 cm^2^, giving a sensitivity of 0.94 and specificity of 0.65. A total of 29 (52%) women had a 3D resting area below this cut-off. Twenty-five (45%) women fulfilled both criteria.

**Table 2:** Hiatal dimensions of nullipara at 20 weeks gestation grouped by mode of delivery with comparison of groups (independent samples *t*-test)

	NVD *n* = 40 Mean (95% CI)	OD *n* = 16 Mean (95% CI)	*p* value
2D AP diameter rest (cm)	5.7 (5.4–6.0)	5.0 (4.7–5.3)	0.007
2D AP diameter contraction (cm)	4.3 (4.1–4.5)	4.0 (3.8–4.3)	0.149
3D hiatal area rest (cm^2^)	18.5 (17.4–19.5)	14.9 (13.8–15.9)	< 0.001
3D hiatal area contraction (cm^2^)	12.8 (11.9–13.8)	11.4 (10.4–12.5)	0.100
3D hiatal area Valsalva (cm^2^)	23.2 (21.1–25.4)*	21.1 (18.1–24.1)	0.260

NVD = normal vaginal delivery; OD = operative delivery; AP = anteroposterior

*n = 39

We found a weak significant inverse correlation between the active second stage of labour and anteroposterior diameter at rest, r = −0.330 (*p* = 0.022), anteroposterior diameter at contraction, r = −0.365 (*p* = 0.011) and area at rest, r = −0.324 (*p* = 0.025) measured at 20 weeks gestation. We found a moderate inverse correlation between the active second stage of labour and area at contraction, r = −0.521 (*p* < 0.001).

## Discussion

We found that a shorter levator hiatal anteroposterior diameter at rest measured with 2D ultrasound and a smaller 3D hiatal area at 20 weeks gestation were associated with operative delivery. The 2D anteroposterior diameter had similar predictive performance for operative delivery as 3D hiatal area with AUC 0.75 and 0.83, respectively. Smaller hiatal dimensions were associated with longer duration of second stage of labour.

Previous studies are scarce and inconsistent but indicate a possible association between antenatal hiatal dimensions and mode of delivery [12, 13]. Lanzarone et al. examined 60 nullipara at 36–40 weeks gestation and found no association between levator hiatal area, anteroposterior or transverse diameter, measured in the 3D volume and delivery mode [11]. Similar to our study, they found a weak inverse correlation between antenatal hiatal dimensions and length of second stage of labour. In a study including 231 women, Siafarikas et al. demonstrated an association between larger transverse levator hiatal diameter and hiatal area measured with 3D ultrasound at 37 weeks gestation and operative delivery [12]. They also found a correlation between larger levator hiatus and shorter second stage of labour. Van Veelen et al. used 3D ultrasound and found that women who delivered by caesarean section because of failure to progress had a significantly shorter transverse diameter and smaller area on contraction at 12 weeks gestation, and a shorter anteroposterior diameter on contraction at 37 weeks, than those with a normal vaginal delivery [13].

The correlation between duration of second stage of labour and hiatal dimensions provides a possible explanation for the association between hiatal dimensions and mode of delivery by mediation. Maternal height was not associated with hiatal dimensions or mode of delivery.

All previous studies used 3D ultrasound, requiring post-processing of the ultrasound volumes. Unique for our study is that we measured the 2D anteroposterior diameter in the midsagittal plane at rest and found a strong association to mode of delivery; 2D anteroposterior diameter had similar predictive performance for operative delivery as 3D hiatal area, but 2D ultrasound is more convenient in a clinical setting since it requires no post-processing of the ultrasound volume. In our study, the mean difference in 2D anteroposterior diameter between groups of normal vaginal delivery and operative delivery was 0.7 cm. The above-mentioned studies have found mean differences of 0.2–0.5 cm for 3D measurements [12, 13]. Whether the larger diameter difference between the groups in our study population is linked to the use of 2D and not 3D ultrasound remains hypothetical.

Apart from using 2D measurements, the novelty of our study is that we constructed ROC curves to test the predictive performance of ultrasound measurements at 20 weeks gestation to identify women at increased risk of operative delivery. When selecting women for low-risk delivery units (or home delivery), it is important to exclude women at increased risk of operative delivery. We therefore suggested a cut-off for anteroposterior diameter of 5.6 cm, giving high sensitivity (0.94) and acceptable specificity (0.63) in predicting increased risk of operative delivery in primipara. For comparison, the cut-off for 3D resting area was set to 17.6 cm^2^, giving a similar sensitivity of 0.94 and a specificity of 0.65, and identifying the same women as the 2D measurement does. Women with hiatal measurements below these cut-offs could probably be counseled not to give birth in maternal wards with minimal monitoring or limited possibilities for obstetrical and neonatal intervention. In countries where home birth is an option, the same rationale could be applied, and perhaps the cut-off should be even higher in this setting.

One of the strengths of this study is the prospective longitudinal design with examination of women before and after delivery. The sonographic methods used are well established, and reliability data from this population indicated good inter- and intrarater validity [14]. The off-line analysis was performed by one examiner, reducing bias. In a clinical setting, 2D ultrasound probes are readily available; the levator hiatal anteroposterior diameter is easy to measure and requires no post-processing. Hence, this measurement could easily be incorporated in a routine mid-pregnancy ultrasound examination. By using measurements obtained at rest, one avoids the need to educate women in pelvic floor contraction and Valsalva manoeuvre, reducing sources of error [17, 18]. Another strength is that we examined participants at 20 weeks gestation, corresponding to the timing of routine ultrasound scan in all Nordic countries. The timing is presumably of importance as the dimensions of the levator hiatus increase during pregnancy [19, 20]. We argue that determination of risk of operative delivery in mid-pregnancy allows clinicians to plan follow-up in pregnancy and select women with need of additional monitoring during delivery. In turn, this could promote more efficient management of maternal wards.

The small sample size reduces power and sub-analysis of vacuum and caesarean section was not possible, nor did we stratify for indication of intervention, i.e., foetal distress or failure to progress. However, it is unclear whether the levator ani muscle plays a role in fetal distress. It has been hypothesized that a poorly compliant pelvic floor may prolong second stage of labour or increase intrauterine pressure and thereby cause foetal distress and increased risk of operative delivery [11]. Our participants were Caucasians, influencing the external validity of the study as muscle biometry differs between ethnic groups [21]. We have not analyzed data on head circumference or foetal head station/position prior to operative delivery. These factors may have influenced birth outcomes.

This study forms the basis for a more comprehensive, power-calculated study addressing the associations between hiatal dimensions measured with 2D ultrasound in mid-pregnancy and mode of delivery. Hiatal dimensions could be included in a framework for risk assessment of operative delivery. In a larger study, it would be possible to study any difference in hiatal dimensions between women with caesarean and operative vaginal deliveries and differentiate between indications for operative delivery (foetal distress and failure to progress) and different ethnicities. One could also explore any relationship between hiatal dimensions and obstetric complications, such as perineal trauma and post-partum hemorrhage.

In conclusion, smaller hiatal dimensions at 20 weeks gestation were associated with longer second stage of labour and increased risk of operative delivery in nullipara. We suggest that women with a resting 2D anteroposterior hiatal diameter < 5.6 cm and 3D hiatal area < 17.6 cm^2^ could be considered at increased risk for operative delivery and monitored accordingly, but we acknowledge that a larger and properly powered study which includes additional obstetric variables is needed to confirm these results.
